# Women’s experiences of receiving care for pelvic organ prolapse: a qualitative study

**DOI:** 10.1186/s12905-019-0741-2

**Published:** 2019-03-15

**Authors:** Purva Abhyankar, Isabelle Uny, Karen Semple, Sarah Wane, Suzanne Hagen, Joyce Wilkinson, Karen Guerrero, Douglas Tincello, Edward Duncan, Eileen Calveley, Andrew Elders, Doreen McClurg, Margaret Maxwell

**Affiliations:** 1 University of Stirling, Stirling, UK; 2 Nursing Midwifery and Allied Health Professions Research Unit, University of Stirling, Stirling, UK; 3 Nursing Midwifery and Allied Health Professions Research Unit, Glasgow Caledonian University, Glasgow, UK; 4 NHS Greater Glasgow and Clyde, Glasgow, UK; 5 University of Leicester, Leicester, UK

**Keywords:** Prolapse, Person-centred care, Shared decision making, Women’s experience, Health services, Women’s choice

## Abstract

**Background:**

Pelvic organ prolapse is a common urogenital condition affecting 41–50% of women over the age of 40. To achieve early diagnosis and appropriate treatment, it is important that care is sensitive to and meets women’s needs, throughout their patient journey. This study explored women’s experiences of seeking diagnosis and treatment for prolapse and their needs and priorities for improving person-centred care.

**Methods:**

Twenty-two women receiving prolapse care through urogynaecology services across three purposefully selected NHS UK sites took part in three focus groups and four telephone interviews. A topic guide facilitated discussions about women’s experiences of prolapse, diagnosis, treatment, follow-up, interactions with healthcare professionals, overall service delivery, and ideals for future services to meet their needs. Data were analysed thematically.

**Results:**

Three themes emerged relating to women’s experiences of a) Evaluating what is normal b) Hobson’s choice of treatment decisions, and c) The trial and error of treatment and technique. Women often delayed seeking help for their symptoms due to lack of awareness, embarrassment and stigma. When presented to GPs, their symptoms were often dismissed and unaddressed until they became more severe. Women reported receiving little or no choice in treatment decisions. Choices were often influenced by health professionals’ preferences which were subtly reflected through the framing of the offer. Women’s embodied knowledge of their condition and treatment was largely unheeded, resulting in decisions that were inconsistent with women’s preferences and needs. Physiotherapy based interventions were reported as helping women regain control over their symptoms and life. A need for greater awareness of prolapse and physiotherapy interventions among women, GPs and consultants was identified alongside greater focus on prevention, early diagnosis and regular follow-up. Greater choice and involvement in treatment decision making was desired.

**Conclusions:**

As prolapse treatment options expand to include more conservative choices, greater awareness and education is needed among women and professionals about these as a first line treatment and preventive measure, alongside a multi-professional team approach to treatment decision making. Women presenting with prolapse symptoms need to be listened to by the health care team, offered better information about treatment choices, and supported to make a decision that is right for them.

**Electronic supplementary material:**

The online version of this article (10.1186/s12905-019-0741-2) contains supplementary material, which is available to authorized users.

## Background

Pelvic organ prolapse (referred to as prolapse) is a common urogenital condition affecting 41–50% of women over the age of 40 [1, 2]. It involves symptomatic descent of one or more of the anterior or posterior vaginal walls, apex of the vagina or the uterus [3]. Common symptoms include a feeling of pressure and vaginal bulging, discomfort in the perineum, pelvic and back pain, and a range of urinary and bowel symptoms including incontinence, sexual difficulties and psychological distress [3, 4]. Although not life threatening, prolapse has a significant impact on women’s physical, psychological and social well-being and quality of life [5]. Urinary incontinence often co-exists with prolapse, adding considerable distress, embarrassment, and discomfort [6].

Prolapse can be managed by lifestyle changes such as losing weight, by pelvic floor muscle training (PFMT), vaginal pessaries, surgery or a combination of these [7–10]. Prolapse treatment decision often depends on the stage and severity of the condition. Early detection of prolapse is important as some conservative treatments such as lifestyle changes and PFMT may prevent aggravation of symptoms and reduce the need for surgical treatment. Evidence for the effectiveness of these treatments is however mixed; no option offers certainty of symptom relief and each carries a mixed profile of benefits, unwanted effects and their probabilities [8–10]. In addition to stage and severity, decisions about treatment also depend on how women value the different benefits and unwanted effects associated with each option and their informed preferences for treatments [4, 10, 11].

Person-centeredness is a crucial element and indicator of the quality of care and is advocated in healthcare policies worldwide [12–14]. It is defined as providing care that is respectful of and responsive to people’s preferences, needs and values and ensuring that people’s values guide all clinical decisions [12]. It requires that people receive the support they need to make decisions and participate in their own care [14, 15]. Despite the importance of person-centred care for prolapse, little is known about the extent to which health services are responsive to women’s needs and what women’s experiences of care are when using the health services for management of prolapse. This study aimed to explore women’s experiences of receiving a diagnosis of prolapse and seeking treatment for prolapse, and to determine women’s needs and priorities for improving the person-centeredness of care in the context of the UK’s National Health Service (NHS). The study formed phase I of a larger study (PROPEL) implementing the delivery of PFMT for prolapse by different professional groups across three NHS UK sites [16].

## Methods

Within PROPEL, the local sites were provided with resources to identify and train staff to deliver PFMT for women with prolapse, with the staffing and format of service delivery being determined locally through a series of service planning meetings in phase 1 of the project. To provide a service-user input to these planning meetings, focus groups with women receiving care for prolapse in local areas were conducted exploring current experiences of services and care, preferences for service delivery models and their visions for a responsive and woman-centred service. This paper reports the findings from these focus groups as they are likely to prove insightful for designing women’s health services internationally.

### Design and sampling

The study used a cross-sectional design with a qualitative approach, drawing on the theoretical lens of symbolic interactionism (a sociological and social-psychological perspective) to understand health behaviour based on the meanings that individuals ascribe to objects and/or actions in their everyday lives [17, 18]. The study was conducted using focus groups and interviews to elicit women’s experiences of seeking and receiving care for prolapse in the UK. Focus groups, rather than individual interviews, were used as they a) offer a more naturalistic setting than being interviewed by a lone and unfamiliar interviewer [19], b) stimulate greater elaboration and re-evaluation of opinions, allowing opportunities to qualify, amplify, amend or contradict stated views through interaction with others [19], and c) have been shown to be more useful in exploring sensitive topics as participants feel less exposed and more reassured by similar concerns/opinions by others [20]. The initial plan was to hold four focus groups across the three sites (one site hosting two focus groups due to the large geographical size of the service area), with a minimum of four participants per focus group. However, participant availability prevented a focus group being held in one location due to remote and rural geographical location and participant ability to travel. Instead four individual telephone interviews were conducted with consenting participants in that area, using the same topic guide as for the three focus groups.

Sampling decisions were influenced by the PROPEL study design which involved a realist evaluation approach using multiple case study design. Sampling was purposeful at the level of NHS sites to maximise variation in service delivery contexts and women’s experiences of service and care within these differing contexts. Three NHS sites were selected purposively to reflect a mix of: urban/rural locations; previous involvement/non-involvement in research into the effectiveness of PFMT for prolapse, namely the UK Pelvic Organ Prolapse PhysiotherapY (POPPY) trial [7]; and current differences in service delivery models (see Table 1). Within the three sites, the sampling frame centred on women’s experiences of receiving prolapse care and needs and priorities for person-centred service. The criteria for potential participants were females > 18 years, seeking and receiving care for prolapse through the local gynaecology/women’s health service between November 2016 and March 2017. Sample size was guided by preliminary data analysis taking place simultaneously with data collection to identify data saturation. No new themes emerged from the data after about three focus groups and two interviews. Data collection was ended when data saturation was confirmed following further two interviews.

**Table 1:** Study site descriptions

Site	Urban/rural	Current dominant model
1	Urban	Primary and secondary care provision of specialist physiotherapy referred by primary care and acute services with a mix of 1:1 and group provision available. Not routinely sent to physiotherapy as first line treatment. Several POPPY trial intervention physiotherapists providing current input to women with prolapse.
2	Urban	Currently has some specialist physiotherapy involvement but greater specialist nurse-led service for women
3a and 3b	Rural	Current interest in re-design and expanding to junior grade physiotherapists and other nursing staff (as practitioners with special interest)

### Participant recruitment

Participants were identified and recruited by either the specialist pelvic floor dysfunction/women’s health physiotherapists or consultant gynaecologists/urogynaecologists in local sites. Staff provided information packs to women in their service containing an invitation letter, study information sheet, consent form, reply slip and pre-paid envelope. Each site had a slightly different approach to the provision of study packs due to the variations in local services, for example, one site had a prolapse education group and provided packs to women at the end of these sessions. At other sites, packs were either provided to women at individual appointments or were sent in the post. Whichever method was used, clinicians were asked to give packs to women in their service currently receiving treatment for prolapse. Women interested in the study returned reply slips to the research team. A total of 240 study packs were sent out by the research team to the three sites but the number of packs actually given out to women by the staff in sites is unknown. Responses expressing interest in the study were received from 39 women. However, practical difficulties of setting up dates, times and venues of convenience to interested participants, partly due to the remote and rural location of the sites, meant that data could only be collected from 22 women. The study was granted ethical approval by the NHS Wales Research Ethics Committee 7 REC ref.: 15/WA/0427 on 27/11/2015.

### Data collection

Women returning the reply slip with an interest in participation were contacted by the research team to ascertain their availability and communicate arrangements for focus groups. All focus groups were conducted in private, closed rooms in local hospitals between November 2016 and March 2017. They were facilitated by two female members of the research team, KS (lead facilitator, an allied health professional and PhD; research fellow on the project, experienced in qualitative and focus group interviewing), and SW (support facilitator, a qualified physiotherapist and PhD, research fellow on project) using a semi-structured approach. Facilitators, not known to participants prior to data collection, made introductions during initial contact and focus groups. At the beginning of discussions, facilitators explained that the purpose of the focus groups/interviews was to explore women’s experiences of prolapse care and their ideals for a person-centred service, which would be fed back to local service planning meetings. A topic guide (see Additional file 1) created by the study team, with input from the study’s public and patient involvement group, facilitated discussions about women’s experiences of prolapse, help seeking, diagnosis, making treatment decisions, receiving treatments, follow-up, interactions with healthcare professionals, the overall service and their ideals for a future service. Each participant provided individual written consent prior to or on the day of the focus group/interview. The focus groups lasted approximately one hour and involved 4, 5 and 9 participants respectively. The telephone interviews lasted approximately 20–30 min. All were audio-recorded and transcribed verbatim, with all transcripts anonymised for women’s names and locations.

### Data analysis

**Fig. 1:**
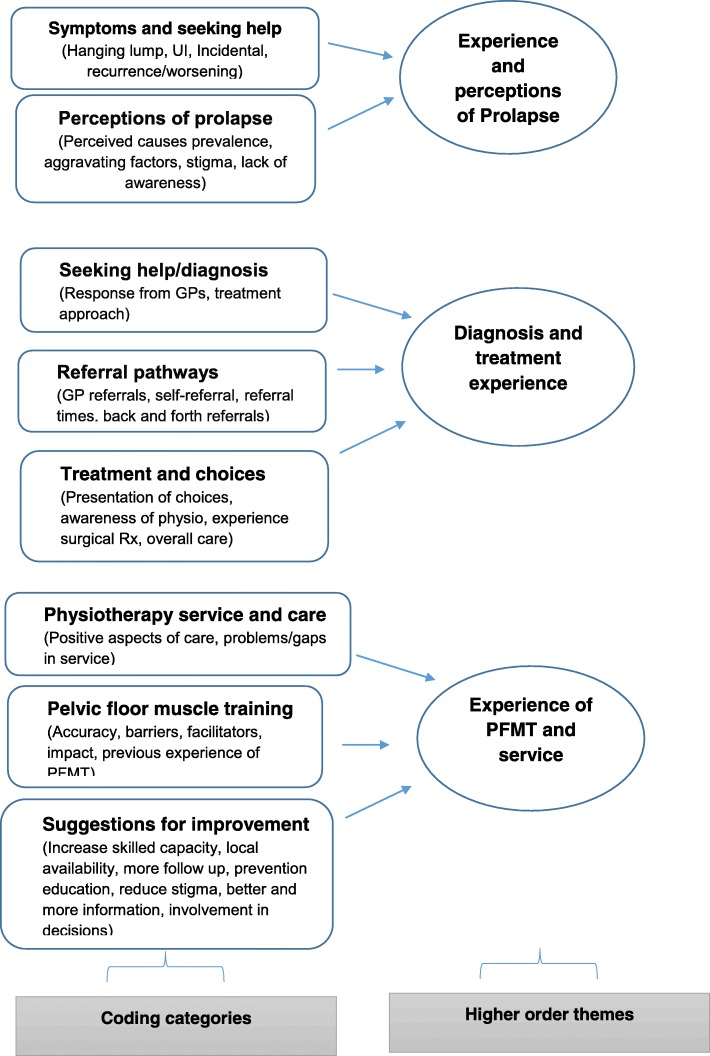
Final descriptive coding framework for focus group/interviews

**Fig. 2:**
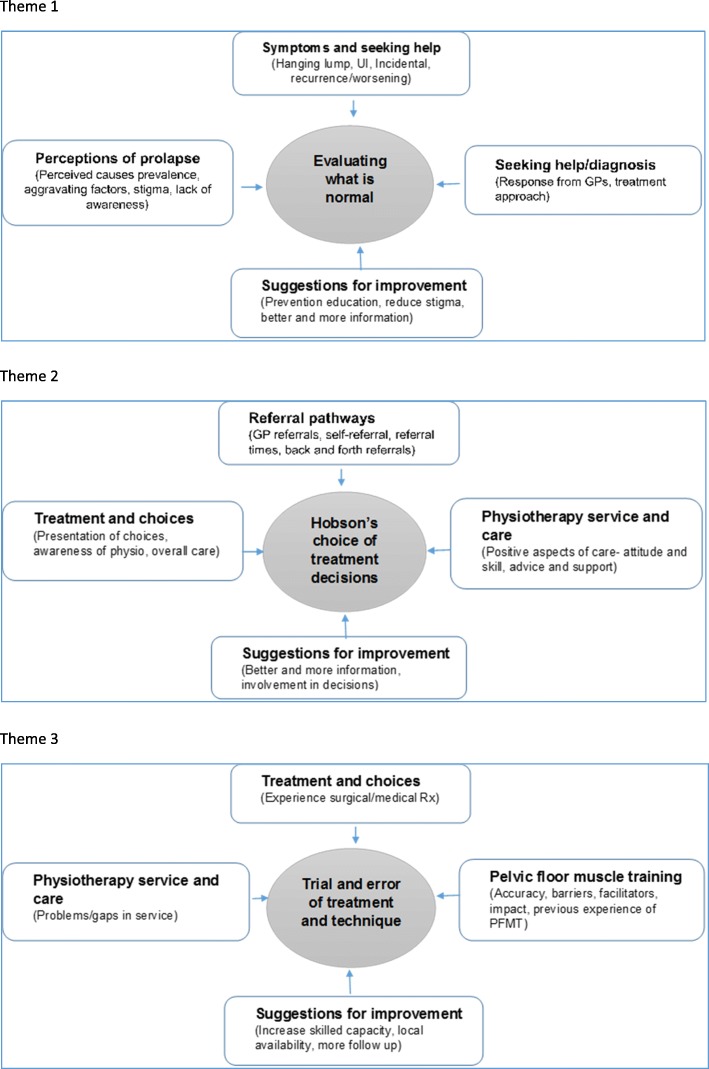
Development of analytical themes from the descriptive coding framework

Data were subjected to thematic analysis driven by our theoretical interest in the area of interactional/communicational aspects of person-centred care [17]. The analytical aim thus was to provide a rich and detailed qualitative account of the ways in which women’s interactions with health professionals and services shape their experiences of care. Using this approach we systematically identified, analysed and reported key patterns within the data relating to the interactional aspects [21]. The analysis was conducted by three members of the team (PA, IU and JW – all experienced health service researchers with experience in qualitative methods) in parallel with the data collection process to allow for data feedback to the local service planning meetings as well as identify data saturation. Data analysis proceeded in following phases [21]: 1) Familiarisation - each transcript from the focus groups and interviews was read and re-read by the researchers to familiarise themselves with the data. To provide feedback to service planning meetings, each dataset was summarised descriptively by PA and JW, noting an initial list of ideas about what is in the data. 2) Generating initial codes – the descriptive summaries of the data were compared, noting similarities and differences in meanings, generating a list of initial semantic codes reflecting discrete segments of meaning, and organising the data into meaningful groups. Codes were both driven from the data as well as informed by our research questions. This initial coding frame was then systematically applied to all the focus group and interview data transcripts using QSR NVivo v11 by IU as a means of managing qualitative data. New codes were added as they emerged through data analysis and were discussed within the analysis team to ensure consistency and validity of interpretation. 3) Searching for themes: Once all data had been coded, the codes were refined by revisiting the text in each category, merging similar codes or moving codes between categories as relevant. Codes were sorted into higher order categories by identifying relationships among different codes and grouping them together. The categories were further grouped under potential themes. This resulted in a refined descriptive coding framework which classified all the data into three higher-order themes, each with a set of categories (Fig. 1). 4) Reviewing themes: Analytical memos were developed throughout the coding process to note recurring themes, inter-relationships between the themes, refuting and conforming data and multiple interpretations of the data. Using these memos, three analytical themes (Fig. 2) were developed which synthesised the data indexed within the descriptive coding framework to address the study’s aims. 5) Defining and naming themes: each theme was considered in detail to identify the story captured within the theme and in relation to the study’s aims. The working titles given to themes during phase 4 were replaced with names reflecting the essence of the meaning contained within them. The three themes were: ‘evaluating what is normal’, ‘Hobson’s choice of treatment decisions’ and ‘trial and error of treatment and technique’. The account of each theme concludes with women’s visions for a person-centred service in light of their experiences. Quotes provided are not intended to be representative of all participants’ views, but are illustrative of data themes.

## Results

Twenty-two women receiving prolapse care through local services took part in three focus groups and four telephone interviews. One participant was Asian, the remaining were Caucasian. All spoke and understood English.

### Theme 1: evaluating what is normal – *‘you are alright, it’s not bad’*

Women varied in terms of the symptoms experienced and when and why they decided to seek help. For some, prolapse was diagnosed incidentally at a smear test. Others reported experiencing symptoms such as ‘leaking’, ‘hanging lump’, and ‘discomfort’ but not knowing the cause, whether they were ‘normal’ and/or common and could be treated. This lack of awareness seemed perpetuated by the stigma and embarrassment around the symptoms which prevented them from disclosing to others or seeking medical help. Consequently women often left the problem to become quite severe before deciding to seek help. General practitioners (GPs) were consulted when symptoms started to affect their daily living, functioning and sleep and cause significant stress and anxiety.

*For me, I was embarrassed to speak to anybody, really, about it, for a long time. But now, I regret that I did that, because I left myself to a bad stage. (site-3a FG)*

*I had a prolapse for a long time, 30 years or something, but I wasn't, like anybody to see me. But when the symptoms is getting worse, I went to the GP. (site-3a FG)*

Many however expressed dissatisfaction with their GP visits as they felt opportunities for early diagnosis and referral to specialist services were missed. GPs were reported to lack awareness about prolapse symptoms and early treatment or referral options. They often dismissed the symptoms as being ‘not too bad’ or ‘a bit baggy/loose’, indirectly reinforcing the stigma, minimising the symptoms and discouraging women from consulting again. Women expressed disappointment that they were ‘not taken seriously’ by the GPs until it got to a point that it was affecting their health in other ways. There was also frustration that they were simply asked to use incontinence pads to deal with co-existing urinary leakage, until the symptoms became severe enough for a specialist referral. Using pads was disliked as they were quite expensive, unattractive and uncomfortable due to sweat and lack of aeration. Some felt that the GPs mismanaged their condition and actually made it worse by referring or treating early. By the time they had suspected prolapse and were referred to specialists, the condition had progressed to the extent that surgery was the only viable option.

*So that’s what bothers me the most is that until I felt that it was…the possibility that it was affecting my health in other ways, it really wasn’t a problem, and nobody considered it a problem. No-one talked about exercises, no-one…because I think what the doctor…it’s not bad, you’re alright, love, it’s not bad, and that’s fine (site-3b FG)*

*The GP didn’t know so she couldn’t have referred me earlier, and by the time she’d done the internal it was too late and so she referred me to the surgery. (site-2 Int1)*

Women attributed their prolapse to lack of general exercise throughout life, higher number of pregnancies, hormonal imbalances, persistent cough, hyperemesis, hysterectomy and ageing. Some were more accepting of prolapse than others, perceiving it to be rather inevitable and permanent. They carried a sense of giving up or being resigned to the symptoms as nothing could be done to reduce them. This resigned acceptance stemmed partly from the dismissive or sometimes disparaging reactions and explanations health professionals gave for their symptoms. For instance,

*…but you’re nearly 50, you’ve had five children, you run a lot, you do a lot of exercise. Perhaps slow down a bit, perhaps don’t do this, perhaps…but it was only because my life…my quality of life had gone down substantially for something that actually turned out to be quite relevant, but the other part of it was that the worry…that actually I’d prolapsed a bit or baggy, as I’ve been called, loose, I’ve been called, all really lovely terms, and that really didn’t matter.(site-3b FG)*

Women felt an urgent need for making prevention education available to women before symptoms start appearing and raising awareness about prolapse, its risk factors and ways to prevent it. Many remembered being advised to do pelvic floor exercises following childbirth but were never trained appropriately in terms of technique, frequency and duration, nor were they supported to continue them lifelong. They suggested emphasising the importance of pelvic floor exercises to women right from the time of childbirth through national campaigns, posters in ladies’ toilets, leaflets in GP surgeries, provisions for post-natal checks and pelvic floor exercises in community groups where women meet. A need was also felt for providing opportunities to share and discuss women’s problems in a safe and relaxed environment to reduce stigma and embarrassment e.g. through drop-in clinics where women could receive a ‘health MOT’ to identify any problems earlier on. To achieve timely diagnosis and intervention, women called for greater awareness and training for GPs on early identification of prolapse symptoms and available treatment options. GPs need to be more proactive and refer directly to pelvic floor muscle training to prevent delay in accessing treatment and prevent symptoms worsening.

*F7: I think, maybe, if you had it more at childbirth. You know, it would help, at this later stage in life. Because I wasn't told anything about exercises, or anything. We were told, you know, and it was in all the leaflets, and blah, blah, but not how to do it properly, or how often you had to do it, and how religiously. Or how important it was.*

*F5: That's when it's really needed. (site-1 FG)*

*F2: If you had a women's centre, where you could speak about everything, sort of thing. Maybe not, you know, you're not just going to go to the prolapse clinic, and speak about that. But maybe you could speak about breast cancer, and how to, you know. And quite an informal setting, because this is quite informal, and quite chatty. (site-3a FG)*

### Theme 2: Hobson’s choice of treatment decisions – ‘*he clearly knew what he thought I needed’*

Women reported diverse experiences of interactions with health professionals in relation to making treatment decisions. The majority seemed to reflect traditional, paternalistic modes of interaction while a few seemed close to shared decision making. Many were unaware of what treatments were available and if they could have a say in the matter. They were often presented with a single option, most commonly surgery, to accept or refuse. Information about the availability of any alternatives or women’s suitability for them was rarely shared, until explicitly asked for by women. Some women were offered different options, but these were not followed by adequate information about the treatments or subsequent discussions about women’s preferences. Health professionals’ own preferences were often reflected in the way treatments were offered; for instance, sometimes surgery was presented more favourably than others, resulting in steering women’s choice towards surgery. Consultants who doubted the effectiveness of physiotherapy, advised that women could ‘try it’ but would eventually need surgery. In contrast, physiotherapy was pushed forward by some as the trend had changed in recent years.

*Facilitator: Do you feel like you’ve had much say in what treatment you’ve received?*

*F2: No, but that’s probably, I'm not one to speak up, you know, there is so much, things often seem a little rushed and certainly the last appointment I had got questions I wanted to ask, but I didn't ask them. We were very late going through and my husband came with me and I just felt as though time was of an essence and he clearly knew what he thought I needed and that was it. (site-2 Int 2)*

*…my first thought was that they’d refer me for surgery again, and of course their thinking now is physiotherapy, more surgery you’re just…the muscles must weakened and they’re going to fix it, but it’ll weaken again. So first time there was no physio input, and this time it’s a big push for physio. (site-3b FG)*

In the perceived absence of any alternatives, women often accepted and commenced the treatment recommended by their doctors; some more reluctantly than others. Nevertheless, women did have preferences or ‘gut feelings’ about what may or may not work for them, which they felt were either not elicited or ignored. Some had preferred to avoid surgery for as long as possible; others wanted to have surgery as all other treatments had failed. There was a sense of discontent when women’s preferences did not match those of the professionals, raising issues of consent and compliance. The vaginal pessaries women were given were often disliked and thought to be uncomfortable and ineffective. Women were often given different types of devices one after another, without monitoring if they stayed in place and worked as expected. Women also had to consider whether and how the treatment fitted in their daily life; e.g. some could not consider surgery as they did not have the support at home for recovery. However, women’s embodied knowledge of prolapse, previous experience of treatments and unique life circumstances and needs were often not heeded when deciding on treatments.

*They sent me to see this lady to fit some sort of contraption, and she brought out this dice which was about two inches square…block on a string, and she spent 15 minutes writhing and tugging and trying to get it there, gave up and said, do it yourself. So I had a go, and I said, there’s no way that is going to fit in there. Well, she said, you’ve had two babies. I said, yes, I have, I said, but your bones soften and everything’s different, your physiology’s different when you’re having babies. I said, that’s not going to go in there, and if it does go in there, it ain’t going to come out, so I'm not using it. (site-3b FG)*

In stark contrast to the above, some experiences of decision making were described as ‘considerate’. The consultants laid down the options, provided information about them, involved women in making the decision and allowed sufficient time to decide. Such an approach allowed women to evaluate the options and their consequences in light of their unique circumstances and preferences.

*Because they were explaining my…they didn’t think I needed major surgery but they said you can have like the tape lift it up, whatever, and I’m like, well, I’m not doing that yet, can we try the other option first? Yeah, sure. So yeah, they did give me a choice. I wasn’t bombarded you’re having this, that’s it, full stop, which is good. (site-2 Int 3)*

Physiotherapists were reported to have a better approach to care than doctors and nurses, likely due to the availability of longer time and more opportunity to explain and discuss. They explained the internal physiology in detail, performed internal examinations, demonstrated correct exercise techniques and offered tips on adherence. Their explanations and suggestions seemed to concur better with women’s embodied knowledge of their physiology and prolapse than those offered by doctors or nurses. This seemed to alleviate women’s worries and give them hope and a sense of control. The most valued aspect of physiotherapy care was the physiotherapists’ person-centred attitude; they were described as ‘caring’, ‘taking more notice of women’, ‘being supportive’ and ‘paying attention to what women say’. The physiotherapy care went beyond the demonstration of accurate exercise technique; they offered additional tips and advice on lifestyle tailored to women’s needs, eased women into care in a way that took away the embarrassment and stigma and boosted women’s confidence about doing the exercises.

*…So then they tried the pessary. Now, I don’t know what was wrong with it, it was alright, it went in, it stayed there, she said, don’t leave the hospital for half an hour, go and get a coffee. If it falls out, come back. I mean, that was the science of it all, if it doesn’t fall out, go home, so off I went, and I read all the blurb, and I thought, I just don’t want this, so then I went to the physio, and in fact I’ve got to say it was a very nice journey. I mean, once again I was quite worried, but she examined me internally and she said, yes, this is what we can do, there’s still something there, we can work on it, it wasn’t a lot, and that’s what I’ve done since, and in fact I’m still wearing the small pessary, which for the minute it’s doing the job, and doing the exercises. (site-3b FG)*

### Theme 3: the trial and error of treatment and technique – ‘*I did everything that you had to do’*

Women’s experiences of different treatments and views about their impact on prolapse symptoms were mixed. For some, surgery worked well and was effective in relieving the symptoms but for others it had little effect. Physiotherapy was felt to be beneficial by some but not by all. For a few, none of the treatments seemed to have the desired effect. Many explained the apparent ineffectiveness of physiotherapy exercises in terms of an incorrect technique. Once women started the exercises with the correct technique, they reported noticing improvements in their symptoms, albeit slowly. The exercises seemed to help them regain control of their life, which previously was dictated by symptoms of prolapse and incontinence. They talked about being able to do the things they could no longer do due to incontinence, e.g. sneeze or cough while out walking, getting out and going further without needing the toilet.

*F3: Well, I felt as if I hadn't been doing it properly for years. I thought I was doing it properly, and didn't realise I wasn't doing it properly. So it was great to know how to do it properly.*

*F2: Yeah, because I've just realised, I've not been doing it properly either. And now, I am doing it properly, I find it's helped with what I'm doing. (site-1 FG)*

*I just feel more comfortable down there, because it felt like the prolapse bit would come out sometimes, and you could actually feel it when you were walking or sitting. Very uncomfortable, and sometimes if you caught it wrong it could really be painful. So now I would say in the last month I’ve not had any of that. It’s not come down anymore even, so I’m like whoa, this is really good. (site-2 Int 3)*

Regular and accurate maintenance of the pelvic floor muscle exercises was unequivocally identified as a major challenge. The main barrier to this was forgetting to do the exercises after some time. Some used telephone apps or alarms as reminders, some paired the exercises with daily activities, and others just did them as and when they remembered. Another barrier was losing the confidence in executing the techniques correctly as there was no ‘checking’ or ‘monitoring’ once discharged from physiotherapy care. Many felt ‘left on their own to struggle’ and ‘unsure of doing the right thing’. Finally, fitting the exercises into the daily routine of busy work and family life and daily stresses was found to be difficult, highlighting the need for physiotherapy treatment to account for women’s personal needs and daily life constraints. Given these barriers, women expressed a strong need for longer-term monitoring and periodic follow-up of prolapse symptoms following the appropriate training in pelvic floor muscle exercises.

*F4: the problem I think is that they then stopped once they thought you knew exactly what to do, and there was no further check, and I felt that…and I then found myself in trouble again, and it became very severe, I’m having an operation sometime this year, and I think that an annual check would have been very beneficial because it would have been picked up, hopefully before it got to the stage it’s reached now where it’s actually affecting my lifestyle and everything. So that’s the main thing. I think the service itself, what the physios do is wonderful, they’re great, but just that suddenly being left on your own, and not knowing whether you’re doing something wrong yourself, that’s causing a problem or if it’s just physically you..*. *(site-3b FG)*

## Discussion

This study explored women’s experiences of seeking diagnosis and treatment for prolapse and their needs and priorities for improving the person-centeredness of the services. The theoretical approach of symbolic interactionism [17, 18] allowed exploration of the phenomena of women’s help seeking and experiences of treatment for prolapse from the women’s perspective; understanding the meanings that women attached to their behaviour. With this lens we were able to understand how women’s own confusion and lack of awareness regarding symptoms, coupled with a sense of embarrassment, prevented women from seeking early treatment. Their diagnosis of prolapse was further delayed by dismissive judgements from GPs and lack of early and proactive intervention. Our approach also elicited women’s preferences for care and treatment; even when offered little or no choice and limited involvement in treatment decision making, their embodied knowledge of prolapse and their unique needs and values were either not discussed or were ignored. Women were aware that health professionals’ own preferences were often reflected in the way treatments were offered, thus subtly influencing (and limiting) women’s decisions. Finally, our approach found that women’s experiences of, and the impact of different treatments were mixed but this led to understanding the process as one of trial and error. PFMT was valued by women as it helped them to regain control over their symptoms, and the mastery of learning the techniques gave back choice and control.

Our findings on experiences of seeking care confirm those reported in previous literature [5, 22–27], but also provide new evidence on the experiences of involvement in decisions around diagnosis and treatment. While some of the barriers for seeking help such as lack of knowledge about symptoms, beliefs about ageing, tendency to minimise the importance of symptoms, feelings of shame and embarrassment, and difficulty talking to others are well documented in previous studies [5, 22–24, 26], our study sheds light on important but often under-acknowledged [25] barriers present within the clinical encounter that add to the diagnostic delays, viz. the dismissive response from primary care professionals, their lack of knowledge of symptoms, prevention and treatment and lack of proactive intervention. Our findings are consistent with previous research suggesting that prolapse treatment decisions are preference-sensitive, i.e. they depend on women’s unique values, preferences and needs [4, 10, 28, 29]. However, our study is also the first to highlight the lack of choice, opportunity and support for women’s involvement in making treatment decisions and the need for more person-centred care.

A key observation across the study’s findings is a disconnect between what women think, feel and know from their experience of prolapse and its treatments and the care they received from primary and secondary health services, which seemed at times to undermine person-centeredness. This disconnect can be explained using the concept of ‘embodied knowledge’ from medical anthropology and sociology, which emerged in response to the predominantly biomedical view of health and illness [30]. Rooted in positivist empiricism, western biomedicine privileges objectivity and neutrality and concerns itself with the objectified bodies of patients rather than the embodied patient as an experiencing person [31]. The health professional is seen as the possessor of ‘expert’ knowledge, which is socially endorsed as being superior over other systems of knowledge [32]. Positioning the knowledge of health professionals as authoritative, consequential, and official, western biomedicine regards it as the only legitimate and appropriate basis for making all decisions about patient care [32, 33]. The concept of embodied knowledge, on the other hand, is concerned with the lived experience of one’s own body. In the context of health, this refers to ‘knowledge which is not distinctly explicit, conscious, mentally representative, or articulated. It is, however, well known by the body or through the body, when it is practised, it is born of experience’ [34]. It consists of unique meanings plus practical personal knowledge that becomes the basis of individuals’ preferences and values [35]. The paradigm of person-centred care legitimises and values people’s lived experience and embodied knowledge and offers it a genuine voice during the clinical encounter [36].

In this study, the disconnect between women’s embodied knowledge of prolapse and the prolapse care they received was reflected in women’s experiences at multiple stages of negotiating decisions about diagnosis and treatment. During diagnosis, women in this study seemed to possess an embodied knowledge of something being wrong with them, although they could not recognise the symptoms as being prolapse. They had put up with the symptoms for a long time, seeking help only when the symptoms started affecting their quality of life. This knowledge was met with dismissive responses by GPs as it clashed with their biomedical model of illness focussing on symptom grades and clinical guidelines, thus leading to missed opportunities for timely diagnosis and treatment. Similarly, women who had lived with prolapse for some time and undergone various treatments had built up a body of personal knowledge and gut feelings around what they felt worked or did not work for them. However, when making decisions about treatment, women’s concerns and preferences based on this embodied knowledge were often unheeded by the consultants and nurses. Some of the treatments and reasoning women were offered seemed to clash with their embodied knowledge of prolapse, which led to some disquiet among women about treatments’ effectiveness. On the other hand, satisfaction was higher when advice and explanations were more aligned to women’s embodied understanding of their physiology.

### Limitations

The study’s limitations include a relatively small number of women recruited from three geographic areas of the UK only, which may have a potential impact on the transferability of findings. However, the study sites were purposefully selected to reflect variations in the UK healthcare system e.g. urban and rural location and models of care and service delivery, which enhances the transferability of our findings to the wider health service context. Additionally, similarity of our findings with those of studies on women’s experiences of prolapse and of seeking care for prolapse from across the world, in Europe [5, 23], America [22, 24, 25, 27] and Asia [26] provides further assurance that the experiences may be transferrable beyond the context of the specific healthcare system involved in this study. Another limitation is that no socio-demographic data were collected from the women who took part in the study, which means that we have no objective information on women’s age, socio-economic status, education and occupation. Finally, the combined use of focus groups and individual interviews for pragmatic reasons may have lowered the homogeneity in the data collection process, with a potential threat to the trustworthiness of findings [37]. However, absence of any observed differences in the type of data collected by each method and convergence of key themes across the two methods may suggest enhanced trustworthiness of findings [38].

### Implications for health service delivery

Our findings highlight several aspects throughout women’s prolapse care journey that need to be addressed to make services more person-centred. First, there is a need for greater awareness and education among women about risks and symptoms of prolapse and availability of PFMT as an early intervention, preventing symptom worsening and need for surgery. Women suggested several ways in which this could be achieved. They called for stronger focus on prevention through appropriate training for women in pelvic floor muscle exercises around the time of childbirth; public health campaigns raising awareness of prolapse risk factors, symptoms and early intervention; and ways of incorporating PFMT into women’s community groups. To reduce the barriers of stigma and embarrassment when seeking help for urogynaecological symptoms, women suggested setting up drop in clinics at health centres which could offer a health MOT on a variety of women’s health issues in an informal and approachable environment. Second, GPs need to be trained adequately in early identification of prolapse and greater awareness of PFMT as a first line of treatment. GPs need to be more proactive and intervene early to prevent aggravation of symptoms. Healthcare professionals generally should value the knowledge and experience women have regarding their prolapse during the diagnosis and treatment processes. Third, prolapse care should be delivered in multi-professional teams to increase availability of a wider range of treatment options and an unbiased and informed approach to treatment decision making. Women should be offered a choice of different treatment options, provided with clear and detailed information about their effectiveness, risks and benefits and should be supported to make a decision that is right for them. As most treatments have a complex profile of benefits and risks, women’s values and preferences about these must be incorporated in the decision making process. Finally, women highlighted a need for regular and long-term follow-up of prolapse to monitor adherence, detect problems, and sustain the benefits of treatments longer-term.

## Conclusions

This study identified several areas which could be addressed by health professionals and services to make prolapse care more person-centred in both primary and secondary care settings. As the treatment options for prolapse expand to include more conservative choices, greater awareness and education is needed among women and professionals about these as a first line treatment and preventive measure. There is a need for a multi-professional approach to prolapse care to widen women’s access to these choices. Women presenting with prolapse symptoms need to be listened to by the health care team, offered better information about treatment choices, and supported to make a decision that is right for them. PFMT and the input of physiotherapists was valued by women but longer term follow-up or annual review would be beneficial for longer term conservative management.

## Additional file

Additional file 1: Topic guide for focus group/interviews. (DOCX 16 kb)

## Abbreviations

FG: Focus Group
GP: General Practitioner
NHS: National Health Service
PFMT: Pelvic Floor Muscle Training
POPPY trial: UK Pelvic Organ Prolapse PhysiotherapY trial
PROPEL: Implementation of an evidence based pelvic floor muscle training intervention for women with pelvic organ prolapse.
