# Supplementary material for: Women’s experiences of receiving care for pelvic organ prolapse: a qualitative study
Source: BMC Womens Health. 2019 Mar 15;19:45. doi: 10.1186/s12905-019-0741-2 (PMC6419797; doi:10.1186/s12905-019-0741-2)
Supplement: Supplementary file 1 — Topic guide for focus group/interviews. (DOCX 16 kb) [file 12905_2019_741_MOESM1_ESM.docx]

**Supplementary file 1: Topic guide for focus group/interviews**

- What has your experience been like of attending the service/receiving treatment for prolapse?

Prompts

- How would you describe your experience of receiving treatment for prolapse?
- How does the treatment service for prolapse work here?
- Did you get much say in your treatment choices, were you made aware of any alternatives that were available to you?
- Could you talk me through how you entered the service?
- What is good about the service?
- Anything not so good/needs improvement?
- What are your previous experiences with PFMT exercises?

Prompts

- How much have you heard about PFMT?
- What have your experiences been of PFMT?
- What do you think of them? Have they been useful? Any downsides/barriers?
- How can the service for prolapse be designed so that it caters for/accounts for women’s needs?
- What might a healthcare professional say to encourage you to try PFMT? Would you be more or less likely to try if it was recommended by certain professional groups? E.g. nurses, physios, specialist physios, GP’s, urologists?
- What might make it easier to attend PFMT services? (location, format of delivery)
- Do you have any other ideas for local planning teams to deliver a PFMT service that is responsive to women’s needs?
